# Perceived barriers to integrated care in rheumatoid arthritis: views of recipients and providers of care in an inner-city setting

**DOI:** 10.1186/1471-2474-12-19

**Published:** 2011-01-17

**Authors:** Louise C Pollard, Helen Graves, David L Scott, Gabrielle H Kingsley, Heidi Lempp

**Affiliations:** 1 Department of Rheumatology King's College London School of Medicine, London, UK; 2 Department of Rheumatology, University Hospital Lewisham, London, UK; 3 National Institute for Health Research, comprehensive Biomedical Research Centre at Guy's and St. Thomas NHS Foundation Trust/King's College London, London, UK

## Abstract

**Background:**

A number of recent reports published in the UK have put the quality of care of adults with Rheumatoid Arthritis (RA) centre stage. These documents set high standards for health care professionals and commissioning bodies that need to be implemented into routine clinical practice. We therefore have obtained the views of recipients and providers of care in inner city settings as to what they perceive are the barriers to providing integrated care.

**Methods:**

We conducted focus groups and face to face interviews between 2005-8 with 79 participants (patients, carers, specialist medical and nursing outpatient staff and general practitioners (GPs)) working in or attending three hospitals and three primary care trusts (PCT).

**Results:**

Three barriers were identified that stood in the way of seamless integrated care in RA from the perspective of patients, carers, specialists and GPs: (i) early referral (e.g. 'gate keeper's role of GPs); (ii) limitations of ongoing care for established RA (e.g. lack of consultation time in secondary care) and (iii) management of acute flares (e.g. pressure on overbooked clinics).

**Conclusion:**

This timely study of the multi-perspective views of recipients and providers of care was conducted during the time of publications of many important reports in the United Kingdom (UK) that highlighted key components in the provision of high quality care for adults with RA. To achieve seamless care across primary and secondary care requires organisational changes, greater personal and professional collaboration and GP education about RA.

## Background

A number of UK national bodies and groups have reported on the components of quality care for people with rheumatoid arthritis (RA). Key recent reports have been published by the National Institute for Health and Clinical Excellence (NICE) [1], the National Audit Office [2] and the King's Fund [3]. These built on earlier reports from the Arthritis and Musculoskeletal Alliance Standards of Care (ARMA) [4] and British Society For Rheumatology (BSR) guidelines [5,6]. These reports overlap with the new focus on quality care throughout the National Health Service (NHS) [7]. Long-term disorders like RA require similar seamless integrated care across the primary/secondary interface as that established for diabetes for example [8].

We report the key findings from an extensive qualitative review of services for RA provided in an inner city environment serving an ethnically diverse and relatively deprived population. Our principal goal was to assess the perceived barriers that prevent the provision of seamless integrated care across the primary and secondary healthcare sectors. We assessed the varying perspectives of patients, carers, specialists and general practitioners (GPs). Studying such a representative and diverse group of patients, carers and clinicians avoids limitations from concentrating on selected patients and clinical staff linked to national groups.

## Methods

### Participants

Focus groups and face to face interviews held between 2005-8 involved 79 participants working in or attending three hospitals and three primary care trusts (PCTs). PCTs were part of the NHS and provided some primary and community services or commissioned them from other providers, and were involved in commissioning secondary care. These groups comprised:

*a. Two patient focus groups: *a purposive sample of 11 RA patients was obtained from one hospital outpatient department; their selection was stratified by disease duration, gender, ethnicity and age. They comprised 8 females and 3 males with a mean age of 58 years and mean disease duration of 12 years; eight were Caucasian and three from black and ethnic groups. One patient refused to take part.

*b. Patient interviews: *a quota sample of 26 patients was obtained from the same hospital as the focus group participants and one other hospital outpatient department to reflect socio-demographic characteristics and duration of illness of the two RA clinics' population. They comprised 22 females and 4 males of mean age 56 years and mean disease duration ten years; 18 were Caucasian and eight from ethnically diverse groups. There was no overlap in the patients between the individual interviews and focus groups. Nine patients refused to take part.

*c. Carers interviews*: the carers consisted of a convenience sample of 11 carers from two hospital outpatient departments. They were approached by staff and the researcher through the RA patients they were caring for. They included five females and six males of mean age 61 years; seven were Caucasian and four from other ethnic groups.

*d. Specialist Health Care Professionals focus group: *six representative members of one multidisciplinary team participated, consisting of consultant rheumatologist, consultant orthopaedic surgeon, rheumatology nurse specialist and allied health professionals (occupational therapist, physiotherapist and podiatrist).

*e. Specialist Health Care Professionals interviews: *15 secondary care specialist staff (6 consultants, 4 specialist registrars and 5 rheumatology nurse specialists) from three hospitals. Eight declined participation in the study.

*f. Generalist Health Care professional interviews: *13 GPs from three local PCTs.

All patients met the American College of Rheumatology criteria for RA. Socio-demographic details of patients and carers are summarized in Table 1. Written consent was obtained from each participant and each study was fully approved by the relevant local Research Ethics and Research and Development committees.

**Table 1:** Patient and Carer Demographics

		Patient Focus *(n = 11)*	Individual Interviews *(n = 26)*	Carers *(n = 11)*
**Female**		8	22	5
**Male**		3	4	6
**Age, mean years (range)**		58 (33-70)	56 (25-85)	61 (36-74)
**Disease duration, mean years (range)**		12 (0.5-32)	10 (1-29)	N/A
**Ethnicity**	**Caucasian**	8	18	7
	**Afro-Caribbean**	3	7	3
	**Asian**	0	1	1

### Data Generation

The audio taped focus groups and 1:1 interviews were carried out in private rooms using a semi structured interview guide [9]. The interview schedules were based on related literature [10,11] and the researchers' experiential knowledge. Focus groups and interviews took between one to two hours.

### Analysis

Interview and focus group information was transcribed verbatim. Qualitative computer software NVivo 8 was used to analyse and handle the data. Content and discourse analysis were applied [12,13], including single counting [14] and deviant results [15]. For validation of the data, external qualitative co-researchers, not involved in the data gathering and analysis, cross-checked initial codes and reached agreement with the researchers about the codes for further data analysis. In addition the data were also presented to two experienced clinicians to assess resonance and plausibility with their clinical experiences. To determine the significance for routine clinical practice in an inner city setting we specifically examined the data from all six qualitative studies for relevance in response to the recent publications.

## Results

Through detailed examination of all the data three main barriers to high quality care were identified. These comprised (i) delayed specialist referral; (ii) limitations to routine follow up and (iii) accessing care in times of need (Table 2). Examples of matters raised by patients, carers and healthcare professionals are summarised in Table 3.

**Table 2:** Summary of Key Themes

Barriers	Non-Professional		Professional	
	***Patients***	***Carers***	***Secondary Care Specialists***	***General Practitioners***

*Specialist Referral*	• Delay in referral from primary care	• Delay in referral from primary care	• Delay in referral from primary care • Quality of referral influences prioritisation	• Role as gatekeepers to specialist care • Need for positive blood tests • Referral linked to personal confidence and role perception

*Routine follow up and DMARD monitoring*	• Lack of time with rheumatologist • Cancellation and postponement of appointments • Perceived lack of knowledge of GPs • Poor primary/secondary care interaction	• Waiting times in clinic • Transport difficulties • Perceived lack of knowledge of GPs • Poor primary/secondary care interaction	• Time pressures • Paucity of follow up appointments • Lack of monitoring by GPs	• Lack of regular review • Lack of clarity of role in monitoring

*Access to care in times of need*	• Difficulty of access during a flare	• No specific comments	• Seeing patients urgently impeded by time pressures and paucity of appointments. • GPs not providing emergency care	• Lack of knowledge of RA treatment • Preference for personal knowledge of rheumatologist to access secondary care urgently

**Table 3:** Quotations Exemplifying Issues In Specific Areas Of Care

Area	Patient/Carer	HealthCare Professional
*Specialist Referral*	I was diagnosed really soon because I put on a lot of weight and at the time I was on a tablet to stop smoking. I thought it was the tablets ....I went straight to the GP and he carried out the rheumatoid blood tests. So I was diagnosed pretty early so I felt quite lucky. (Patient 32)	'If they think it's an inflammatory arthropathy, most GPs will send it to the appropriate people, maybe not quickly enough.' ( Consultant Orthopaedic Surgeon)
	'I used to go to the gym so it took my GP nearly a year and a half to find out that I had RA and it was only because I demanded an x-ray... (Patient 37)	'So initial diagnosis is key and quick referral putting the right things in the letter so that when we get the letter we can see what they [GP] think it is...there's huge variation then in that.' (Consultant Rheumatologist)
		'If you don't do the blood test the hospital would be absolutely overwhelmed. If everybody [patient] who thought they might have rheumatoid we refer to hospital, the system would grind to a halt... (GP1).
		'So we normally do blood tests like RA Factor and antibodies and when they come back and yes the suspicion is that they might have RA, then we refer' (GP2).

*Routine follow up*	'Especially if it's a period of time like 6 months [between appointments], you know that's a long time...they should give you a thorough examination, send you for x-rays or god knows what, just to see how you're coping.' (Patient 33)	'It depends what they come in with. You don't always have time.... I mean if they have everything at once it's really difficult to address every issue in that time slot. '(Nurse Specialist)
	'He tries to help me, he is a really understanding doctor. He understands how I feel. I can really talk to him. He knows how I feel. I tell him where I am having the pain. I relate to him.' (Patient 22)	'I think the follow-up in rheumatoid has changed a bit in the last of couple of years from our perspective in that with the pressure on follow up slots being so great, the interval between follow-ups is much longer and it will be pushed out to 6 months or a year' (Consultant Rheumatologist)
	'I have to wait a long while to see the doctor when I got an appointment for a certain time. I have waited one hour and a half, you never go in at the appointment time'. (Patient 15)	'I'd like the GPs to take on blood monitoring, I think that is a complete waste of time for us to look at each single blood result for 100 of 100 of patients and so I would like to have blood monitoring with our support to be out in the community. Patients would prefer that as well'. (Rheumatology Specialist Registrar)
	'I think sometimes the specialists haven't got the time to give you that long chat that you need, whereas the nurse will. You know, not that the specialist doesn't want to...' (Patient 31)	'... so we sort of monitor them [RA patients] from the practice... just doing their bloods, seeing everything is in order and there is no sort of active flare up or anything and we are happy to do that if we get a sort of proper protocol and guidelines in which we can work' (GP 10).
	'My GP, I have... I think I have lost respect...he hasn't really served me particularly well. I have to 'play act' when I see a GP. So I have to pretend that I am really ill and about to die before anything actually happens ... I don't have a lot of faith in them'. (Patient 24)	'.... there is no financial incentive and actually I don't agree with the financial incentive, but if you suffer from an illness that is not included in the QOF, I think there is a degree of neglect and ahm... there is no motivation of the practice to think about that [RA]' (GP 4).
	'Well my GPs quite good. If it wasn't for my GP half the things he told [advised] me what to do. If you come to the hospital, you ask how you do this, nobody tells you.' (Patient 30)	
	"In the end I got so mad with them (GP) I started shouting and I said " you know, I've got to come up here and ask you for blood forms every month!"(Carer 8)	
	'Doctors... I think they get tired of me (carer) when I attend the consultation.' (Carer 5)	
	'I mean obviously the waiting times can get on your nerves.' (Carer 11)	

*Access to care in times of need*	'If the nurse thinks I am not all that good, she calls the doctor... and he will come and see me right away' (Patient 1)	**'**.....I like the patients to have better support...seeing someone in six months...is not very helpful...they [patients] just struggle on... I have no follow up appointments...the GPs don't know what to do you see, so it is a dreadful situation... we are under-resourced. I don't think that is the way to deliver it [health care]... knowing that we can't actually do that [provide emergency cover]'. (Consultant Rheumatologist)
		'Patients can now 'choose and book' any hospital which is extremely confusing for GPs,... because I think it is extremely important that the patients go to local hospitals and I am familiar with the consultants and the system there.' (GP 9).

### Specialist Referral

#### Patients and Carers

Most patients (29/37) consulted their GPs when their symptoms started. Many (14/37) reported frustration at delays in specialist referral; only one patient commented on being referred early. Some patients (4/37) reported specialist referrals depended on positive blood tests, two of these patients had been diagnosed within the previous twelve months. Delayed referral was mentioned by few carers (2/11).

#### Healthcare Professionals

All rheumatology specialists in the focus group (3/3) commented on delayed referrals, noting variations in the timing and quality of referrals. This issue did not feature in interviews with individual specialists. Some GPs (4/13) emphasised the need for early referral but many (11/13) commonly waited for 'positive blood tests' for rheumatoid factor before referring. Some GPs (5/13) also waited for confirmatory responses to initial treatment with anti-inflammatory drugs and steroids. A number of GPs (5/13) were influenced by their perceived role as 'gate keepers' to specialist care.

### Limitations in follow up

#### Patients and Carers

Many patients (18/37) commented on the importance of monitoring their RA, highlighting the need for physical examinations together with explanations of disease progress and joint discussion of options of new treatments. They wanted the opportunity to participate in decisions about their care. Patients (12/37) focussed on the value of understanding approaches by staff and developing trusting relationship over time with nurses and doctors.

Some patients (8/37) commented critically about insufficient time during consultations with rheumatologists. By contrast there were many positive comments about interactions with rheumatology nurses (32/37). Patients felt more comfortable discussing matters with specialist nurses, who both understood their concerns and had more time (7/37).

Patients reported organisational problems including long waiting times in clinic (13/37), blood sampling for disease modifying anti-rheumatic drugs (DMARDs) monitoring (9/37), between appointments (5/37) and clinic cancellations and postponements (2/37).

They described mixed experiences with their GPs. On the one hand many (21/37) expressed criticisms about GPs' perceived lack of knowledge of RA and its up-to date treatment (9/37). On the other hand a substantial number of patients reported positive experience in primary care (14/37), often mentioning sympathetic ongoing relationships (8/37). A minority preferred to receive most care in hospital (6/37).

Carers voiced also concerns about waiting times in the clinic (5/11), perceived limited benefits from treatment (4/11) and difficulties with transport to the clinic (3/11). One carer was concerned about GPs limited knowledge. Most (6/11) commented on the importance of good interactions with outpatient clinic staff. Carers noted that RA had major impacts on themselves as well as on the patients they were caring for.

#### Healthcare Professionals

Specialists' views (16/18) echoed patients' experiences about the paucity of follow up appointments and lack of time during consultations; they found it difficult to adopt a holistic approach with patients. They noted that these pressures had resulted in appointments with rheumatologists being replaced by specialist nurse led clinics.

Most GPs (10/13) commented on their role in providing repeat prescriptions after the initial referral of patients with RA, otherwise they are only marginally involved in ongoing care. Only a minority (4/13) reported they regularly reviewed patients with RA. GPs believed they should combine clinical, administrative and emotional support for RA patients, as part of their comprehensive long-term care.

Few GPs (4/13) commented on the negative impact of the Quality and Outcomes Framework (QOF). They stated it influenced their approach to chronic disease management and, as RA falls outside this framework, they thought it reduced the priority given to RA patients in primary care.

### Access to Care in Times of Need

#### Patients and Carers

Patients emphasized the importance of immediate help and support during times of flare of their RA and/or emotional stress (14/37). They tend to approach rheumatology nurses first to gain access to specialists during flare ups. Carers did not comment on this topic.

#### Healthcare Professionals

Most specialists (11/18) agreed that patients need immediate access during an exacerbation of RA and that the service should respond quickly and effectively. However, such access increased pressure on appointments leading to overbooked clinics and long waiting times.

Most GPs (8/13) considered an important pre-requisite for accessing secondary care was having a personal relationship with the consultant(s) and having knowledge about him or her. These professional links helped access to specialists during acute episodes of RA (9/13). When such links did not exist (4/13) it limited successful primary/secondary care integration. One GP thought this relationship was hindered by the 'choose and book' system as patients might be seen in hospitals unfamiliar to them. (Choose and Book is a national electronic referral service which gives patients a choice of place, date and time for their first outpatient appointment in a hospital or clinic.)

## Discussion

This qualitative study identified three key areas in which there were perceived barriers to seamless integrated care in RA from the perspective of patients, carers, specialists and GPs. These are early referral, limitations of ongoing care for established RA and management of acute flares. The study took place during a period in which NICE guidelines and other UK care strategies were being developed [1-7], and therefore helps place our findings in context. Our results are relevant as there are few multi-perspective studies in rheumatology [16] and the multiperspective qualitative approach is very useful to capture the experiences of all stakeholders involved in the treatment and care [17-20].

This qualitative study was conducted in three out-patient clinics and three PCTs consisting of 79 participants of whom 37 were patients. It is difficult to assess how generalisable the findings of this study are, although patients were selected from two different clinics. The question over whether the emerging themes are general ones or merely represent local issues is difficult to answer although evidence from previous studies would suggest that similar issues occur more widely, examples include previous studies which showed delays in referral caused by the presence or absence of positive blood results [21,22], the need for good access and working relationships with specialists [21,23] and the lack of experience/knowledge of the primary care physician [23,24]. The lack of time with rheumatologists and lack of communication between primary and secondary care has also been noted in other parts of the world [23].

Qualitative approaches allow patients to give first-hand accounts of their experiences, in this case their experience of the care provided in primary and secondary settings. By focusing on detailed descriptions and their meaning, such in-depth accounts, from semi-structured interviews, may uncover aspects that cannot be readily captured by structured questionnaires and provide information that is helpful when trying to re-organise services. To our knowledge, no other paper has been published which addresses the views of all stakeholders involved in the care of RA patients.

Delay in referral was highlighted in our study, this has also been suggested in previous guidelines, and observational studies from the UK [2,3]. Experience with both the Norfolk Arthritis Register [25] and the Steroids in Very Early Arthritis trial [26] have shown that it is possible to see UK patients with inflammatory arthritis in the early stages of their disease. The possible causes of delay in referral are complex and there may be several explanations, such as reflecting organisational aspects; however, alternate explanations may include patient issues such as the disparity between actual observed and perceived time to referral in those patients with long disease duration, who may find it difficult to accurately estimate any delays after such long periods. Patients may also take some time to identify their symptoms and hence achieve referral, which may be reflected in a perceived delay in referral. Finally we do not know if many patients present with features that could be interpreted as leading to rheumatoid arthritis but, over time, melt away and do not progress. Other publications have suggested that people with inflammatory arthritis delay seeking medical advice [2] which could also impact on the time to referral to secondary care. In particular previous studies have shown that ethnicity may play a part in the delay in people seeking help [27] as well as their willingness to accept aggressive treatment [28]; these observations are pertinent given the multicultural population served by South London. However, the issue of people delaying seeking help was not discussed by our patients or GPs. One clear message from our research with GPs was that they are concerned about their role as "gatekeepers" to secondary care. This potentially creates reluctance to refer patients with possible inflammatory arthritis for specialist advice and is a barrier that needs to be removed [2].

## Conclusions

There are several limitations in the ongoing management of established RA that could be overcome by changes in the arrangements of the service. One major issue is insufficient time in secondary care appointments so that clinicians do not fully address major concerns for patients. The greater involvement of specialist nurses has been particularly helpful [29], but is not enough by itself. The evidence suggests that specialists should devote more time and resources to the follow up of patients with established RA [10]. The NHS Musculoskeletal Framework [6] should assist this goal by transferring stable musculoskeletal disorders to community based units and allowing specialists to focus on managing RA. This will require a re-evaluation of new to follow-up ratios as low ratios, often considered a mark of effective care, may actually indicate poor quality care in RA.

A second important issue is the limited knowledge many GPs have about RA [3,23,24]. This reflects not only the absence of musculoskeletal disorders from the Quality and Outcomes Framework but also the dearth of rheumatology teaching in the postgraduate training of UK GPs [2]. Whilst some GPs provide high quality care, this is by no means universal [3]. It is impractical to equip all GPs with enough expertise to make significant inputs into the management of RA patients, and the best solution may be to make better use of those GPs with particular expertise in the field. This has been utilised in some parts of the country by the establishment of so-called GPSIs (GPs' with a specialist interest). Many of these GPs could be trained within rheumatology departments running clinics alongside consultants gaining greater insight and knowledge, which can then be transferred into the community setting.

The final key issue is the need for close collaboration between primary and secondary care. Terminology may hinder improvements of service as the distinction actually lies between specialist and generalist. As RA is relatively uncommon and GPs have limited knowledge about the disease, we consider its care needs to be managed by specialists. However, there needs to be better links between specialists and the community they serve and good working relationships between GPs and specialists and this might be better served by basing specialist services within the community. Better professional relationships could also be established by inviting community services into specialist centres to meet specialists and to organise teaching sessions. These ties would need to be continually maintained and would require commitment from both sides as it is unlikely that monetary resources would be available through the NHS although other sources could be sought. However, RA patients often need direct access to X-rays and other specialist opinions. Exact solutions would have to be determined at a local level depending on issues such as travel for patients and local community facilities. We realise that this will be a controversial matter that cannot be readily resolved.

## Competing interests

The authors declare that they have no competing interests.

## Authors' contributions

LP helped to conceive and designed the study, assisted in the data analysis, drafting the manuscript and overseeing the submission process. HG conducted individual interviews with carers and contributed to the data analysis. DS conceived of the study and helped draft the manuscript. GK participated in the design and coordination of the study and helped draft the manuscript. HL helped to conceive and design the study, conducted individual interviews with patients, medical and nurse specialists and GPs and focus groups with patients and specialist multi-disciplinary team members, contributed to the data analysis and helped draft the manuscript. All authors read and approved the final manuscript.

## Pre-publication history

The pre-publication history for this paper can be accessed here:

http://www.biomedcentral.com/1471-2474/12/19/prepub
